# Pathway activation profiling reveals new insights into Age-related Macular Degeneration and provides avenues for therapeutic interventions

**DOI:** 10.18632/aging.100711

**Published:** 2014-12-22

**Authors:** Evgeny Makarev, Charles Cantor, Alex Zhavoronkov, Anton Buzdin, Alexander Aliper, Antonei Benjamin Csoka

**Affiliations:** ^1^ Insilico Medicine, Inc, ETC, Johns Hopkins University, Baltimore, MD 21218, USA; ^2^ Boston University, Boston, MA 02215, USA; ^3^ Retrotope, Inc, Los Altos Hills, CA 94022, USA; ^4^ The Biogerontology Research Foundation, London, UK; ^5^ Pathway Pharmaceuticals, Ltd, Hong Kong, Hong Kong; ^6^ Vision Genomics, LLC, Washington, DC 20010, USA; ^7^ Epigenetics Laboratory, Dept. of Anatomy, Howard University, Washington, DC 20059, USA

**Keywords:** AMD, Age-related Macular Degeneration, gene expression, Transcriptome profiling, signaling pathway activation strength

## Abstract

Age-related macular degeneration (AMD) is a major cause of blindness in older people and is caused by loss of the central region of the retinal pigment epithelium (RPE). Conventional methods of gene expression analysis have yielded important insights into AMD pathogenesis, but the precise molecular pathway alterations are still poorly understood. Therefore we developed a new software program, “AMD Medicine”, and discovered differential pathway activation profiles in samples of human RPE/choroid from AMD patients and controls. We identified 29 pathways in RPE-choroid AMD phenotypes: 27 pathways were activated in AMD compared to controls, and 2 pathways were activated in controls compared to AMD. In AMD, we identified a graded activation of pathways related to wound response, complement cascade, and cell survival. Also, there was downregulation of two pathways responsible for apoptosis. Furthermore, significant activation of pro-mitotic pathways is consistent with dedifferentiation and cell proliferation events, which occur early in the pathogenesis of AMD. Significantly, we discovered new global pathway activation signatures of AMD involved in the cell-based inflammatory response: IL-2, STAT3, and ERK. The ultimate aim of our research is to achieve a better understanding of signaling pathways involved in AMD pathology, which will eventually lead to better treatments.

## INTRODUCTION

### Background

Age-related macular degeneration (AMD) is a major cause of blindness in older people in developed countries, where increases in average age and falling death rates are contributing to the prevalence of the disease [1]. As with many age-related diseases, the main risk factor for AMD is, of course, aging [2] but it also affected by other risk factors like genetics, patient history, smoking, trauma, etc. [3]. With over 196 million people projected to be affected worldwide by 2020 [4], there is an urgent need to discover working therapeutic solutions for treatment and prevention [5, 6]. Clinically, AMD is in fact a set of complex multifactorial diseases caused by the degeneration of the photoreceptors (rods and cones) and retinal pigment epithelium (RPE) cells of the eye in older age [7]. It is the most common cause of visual impairment in the elderly (>60 years) and is broadly classified into two clinical categories, namely: the wet form (neovascular or exudative) and dry form [8].

Both types of AMD pathology start with the formation of insoluble aggregates, drusen, which forms in the extracellular matrix between Brush's membrane and RPE. Drusen consists of complement components, lipo-proteins, cell debris, oxysterols, oxidized phospholipids and RNA. Accumulation and extension of this drusen leads to nutrient and oxygen starvation of retinal and RPE cells. As a result, retinal cells degenerate in the “dry” form of AMD; but sometimes the RPE cells stimulate angiogenic factors (e.g., VEGF, TGFB etc.) which act on the choriocapillary network above the Bruch's membrane and stimulate proliferation of new blood vessels in the areas where blood vessels are not normally present. These newly-formed blood vessels cause disruption of RPE cell integrity, causing the “wet” form of AMD.

### Molecular alterations in AMD

Conventional methods of genetic analysis have yielded important insights into AMD [9, 10]. Specifically, several groups of genes have been identified: complement proteins (factor H, C2, C3), lipid metabolism proteins (CEPT, APOE, LIPC), and angiogenic factors (VEGF family). Interestingly, it has been shown that AMD pathogenesis is associated with lipid metabolism - drusen is comprised (by dry weight) of 3.2% long fatty acid esterified and non-esterified cholesterol and apolipoproteins (apo E and apo B) [11]. Also, a variant of the lipase gene (*LIPC*) (rs493258) is linked with AMD [12]. An explanation is that lipids accumulate with the advancement of age, which may lead to the creation of hydrophobic barrier in the Bruch's membrane contributing to disease pathogenesis. There is strong evidence that APOE single nucleotide polymorphism (SNP) variants can have a differential impact on AMD pathology: for example the APOE2 variant is more common in individuals with AMD compared to the APOE4 variant, as it is believed to be unable to form dimers [13]. Polymorphism of another lipid metabolism protein, CEPT, might also be involved in AMD progression [14], and 7-ketocholesterol, one of the oxidized components of cholesterol metabolism, has also been found to stimulate AMD pathological mechanisms such as angiogenesis and inflammation [15].

### Altered signaling in AMD

Besides lipids, it has been discovered that the dys-regulation of complement pathways is further responsible for AMD pathology [16]. Intriguingly there is a close connection between cholesterol metabolism and the alternative complement pathway. Cholesterol-dependent complement activation is associated with C5/C3 conversion into cholesterol crystals, which consequently induce alternative complement cascades. Also, the accumulation of lipofuscin, with several oxysterols, has been shown to amplify CFH activity along with increased C3b, suggesting lipid intermediate-based complement dysregulation [17].

Lipid metabolic intermediates can also directly affect expression level of genes involved in the inflammatory response. For example recent studies demonstrated that oxidized phospholipids can change the expression of CCR-2, IL-6, IL-8, CD-36 and VEGF, and induce endoplasmic reticulum stress via various NFkB pathways [18]. Moreover it has been shown that in atherosclerosis the regulation of synthesis of inflammatory factors like TNF-α, IL-8, IL-6 etc. in macrophages is mediated by the MEK/ERK pathway [19]. Therefore, to summarize: proinflammatory processes induced by accumulated lipid/cholesterols intermediates play an important initial role in AMD.

### Choroidal neovascularization

Choroidal neovascularization (CNV) also contributes to the severity of AMD pathology in the “wet” form, and is usually caused by VEGF and other pro-angiogenic molecules like TGFB and pro-inflammatory cytokines. CNV might be a way to compensate for a lack of oxygen and nutrients with new vessel formation [20]. Plus the expression of VEGF can be influenced by components of the complement pathway; specifically the expression level of VEGF can significantly increase if C3a/C5a is added to RPE cell culture [21]. TGFb can also form a heteromeric complex with type II *TGF*-beta receptors and mediate TGF-beta signaling. It has also been shown that activated complement components with oxidized sterols and 7-Ketocholesterol (7-KCh, a proinflammatory oxysterol), can directly influence the expression of angiogenic and proangiogenic molecules [22]. Deposition of 7-KCh can directly induce VEGF signaling [23] and induce inflammation and apoptosis of RPE cells mediated by the mitochondrial apoptotic pathway [15].

### Summary of known changes

To conclude, AMD originates from at least 3 groups of signaling pathways: lipid metabolism (in the preclinical stage) – dysregulation of these pathways leads to accumulation of insoluble deposits; complement pathways (preclinical and clinical stage) – aberrant activation of these pathways leads to drusen formation, their extension and subsequent photoreceptor degeneration; and angiogenesis pathways (clinical stage) – abnormal activation of these pathways leads to neovascularization, and new blood vessel outgrowth into the RPE area.

### Development of a new bioinformatics tool: AMD Medicine

Improved understanding of these signaling pathways in AMD has already paved the way for new therapeutic solutions [24-26], but we need a *much* deeper and integrated understanding to fully decipher its true pathogenesis. To this aim we developed a new bioinformatic program called OncoFinder [27, 28]. Based on large-scale transcriptomic data, this novel approach enables quantitative measurements of intracellular signaling pathway (ISP) activations in many cells/tissues and diverse physiological and pathological conditions, including cancer. OncoFinder operates similarly to another recently published approach termed Pathifier [29], which also quantitatively analyzes the extent of signaling pathway activation basing on gene expression data. However, the Pathifier algorithm utilizes different mathematical formulae for calculation of pathway activation scores, and does not take into account specific roles (stimulatory, inhibitory, ambivalent, unknown, etc.) of individual gene products forming a pathway, which may produce a biased output. In OncoFinder, we use a manually curated database of molecular signaling pathways that includes the functional roles present in a pathway [27, 28].

Signaling pathways regulate all major cellular events in health and disease [30-33], and OncoFinder calculates a quantitative measurement of the signaling pathway activation termed “pathway activation strength” (PAS) for the ISPs under investigation. PAS measures the cumulative value of perturbations in a signaling pathway, and may serve as a distinct indicator of pathological changes in the intracellular signaling machinery at the cellular, tissue, or organ level. In previous studies we confirmed the robustness of this approach and its applicability to analyzing intracellular signaling [34]. The PAS calculation algorithm dramatically diminished the discrepancies between the microarray and deep sequencing data obtained using various experimental platforms [27]. The PAS value itself may serve as a new type of biomarker that can distinguish between the pathway activation profiles in different tissue types [28] and was established as a robust biomarker of bladder cancer [35]. The intimate interplay of tissue-specific signaling pathway activation in AMD with age may shed new light into other age-related diseases and eventually aging itself [36, 37]. In this study, we altered and modified OncoFinder so that it is capable of identifying changes in AMD, and renamed the software “AMD Medicine”. It was then used to compare the transcriptomes of normal RPE-Choroid and AMD affected RPE-Choroid tissues. The purpose of this research was to perform for the first time a large-scale profiling of signaling pathway activation signatures in AMD. Our results clearly demonstrate activation of immune, inflammatory and cell proliferation signaling pathways along with down-regulation of apoptotic signaling pathways.

As stated, pathway activation analysis was performed using “AMD Medicine” developed by Vision Genomics LLC using signaling pathway activation analysis algorithms (SPAS) [35]; we picked two sets of transcriptome profiles generated from normal and AMD affected RPE-choroid human tissues. The first dataset, GSE50195, evaluated gene expression levels in 9 human donor eyes with early AMD, and 7 control human donor eyes using the Affymetrix Human Exon ST 1.0 arrays. The second dataset, GSE29801, evaluated gene expression levels in 31 normal, 26 AMD, and 11 potential pre-AMD human donor eyes using the Agilent-014850 Whole Human Genome Microarray 4×44K. Raw data files from the GEO NCBI website were pre-processed using R Bioconductor.

We identified 29 differently activated pathways in RPE-choroid AMD phenotypes. We found a strong correlation between transcriptional profiles established in two independent experiments. 27 pathways were activated in the AMD state compared to the normal state, and 2 pathways were activated in the normal state compared to the AMD state. In AMD, we identified a graded activation of pathways related to wound response, complement cascade, and cell survival. Also, we noticed down-regulation of two pathways responsible for apoptosis. Significant activation of pro-mitotic pathways is consistent with dedifferentiation and cell proliferation events which occur early in the pathogenesis of AMD. Significantly, we discovered new global pathway activation signatures of AMD. These pathways could be considered to be involved in AMD pathogenesis and are involved in the cell-based inflammatory response, which is a hallmark of AMD etiology (IL-2, STAT3, ERK). Our data are in agreement with previously published reports on AMD pathology and provide new insights into molecular etiology of AMD and its future treatment strategies.

## RESULTS AND DISCUSSION

### Background and data sourcing

Even though our understanding of the genetics of AMD has progressed continuously in the last decade, many basic questions still need to be answered. Therefore, in this study, we evaluated changes in functional pathway networks using changes in gene expression of single genes between AMD and normal eyes, as building blocks to reconstruct changes at the pathway level. We also attempt to integrate the pathways generated by our pathway activation analysis into coherent interacting subgroups, and also propose a hypothetical mechanism for how such interactions may occur. In our research, we analyzed in-depth previously published gene expression data publicly available through the NCBI GEO bioinformatics repository website (http://www.ncbi.nlm.nih.gov/geo/query/acc.cgi?acc=GSE50195, http://www.ncbi.nlm.nih.gov/geo/query/acc.cgi?acc=GSE29801). For the first time, we applied the analysis of signaling pathway activation strengths to the transition from a normal state to the AMD state of RPE-Choroid tissues.

### Previous limitations

In previous reports, one of the serious limitations of direct interpretation of microarray gene expression data of AMD-affected tissues was a lack of significant differential expression signatures between the case and control samples for the individual gene products [38, 39]; as a result it was necessary to use expression correlation methods to identify gene expression changes in the data sets with p < 0.1 and a 0.25-fold change [40], or to use clustering algorithms to identify network patterns [41], which are both statistically weak. Particularly, the authors of one of the data sets used in this study (GSE29801) tried to overcome the complexity of the gene expression changes obscured by noise by using cluster analysis to reveal differential gene expression programs, followed by functional enrichments [42]. To overcome these problems we took advantage of a much more robust method of pathway activation analysis [28]. Also, for our analysis we selected only PRE-choroid tissues, since only this data was available in both AMD datasets under investigation.

### Changes in functional pathway networks

**Figure 1:**
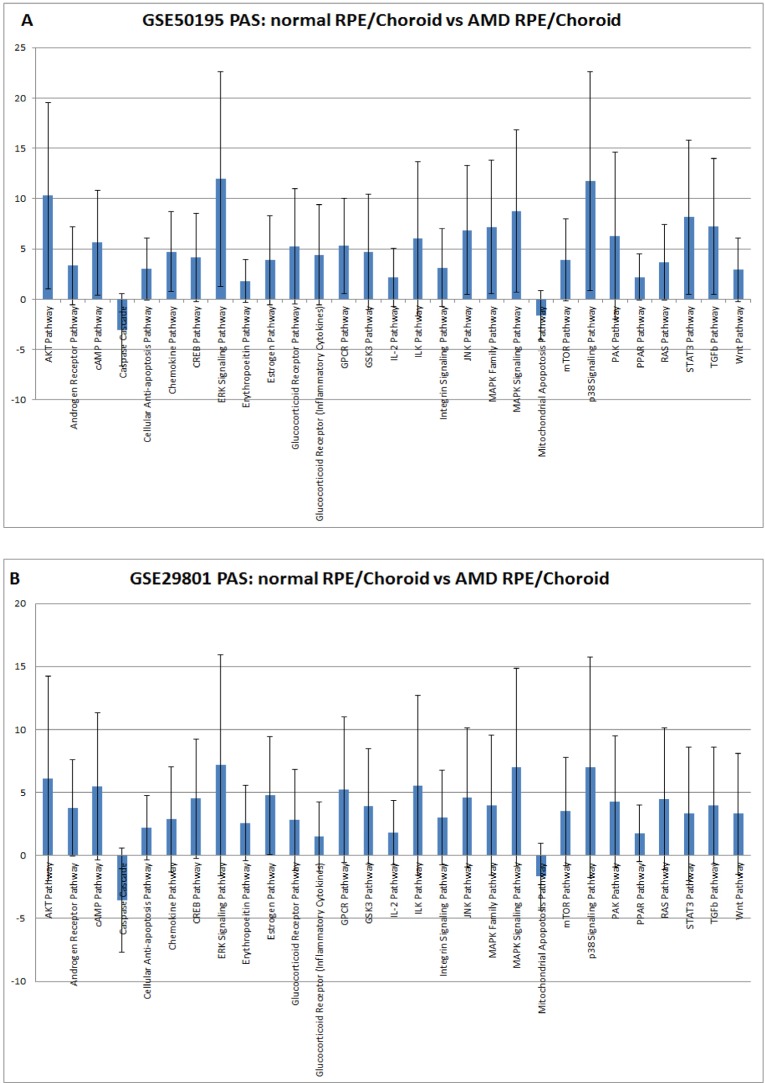
Pathway activation strength (PAS) for selected pathways PAS values have been calculated according to OncoFinder algorithm. PAS presented on this figure passed the following filters PAS<−1.5 and PAS>1.5 in both datasets. Blue bars represent PAS average for each pathway, and error bars represents standard deviation A. PAS derived from GSE50195 dataset. B. PAS derived from GSE50195 that cell-based inflammatory responses within the RPE-choroid are a core feature of AMD. However, cellular sources and targets of pro-inflammatory secreted factors are still need to be determined along with the regulatory mechanism of the chemokine network.

Our method of pathway activation analysis instantly revealed substantial changes in several major pathways in AMD which were previously invisible at the gene expression level. For example, two genes, KDR and FLT1, which belong to the VEGF pathway, demonstrated lower expression in AMD than in controls [39]. We did not find significant changes in VEGF pathway status, but we did find strong activation of major pro-angiogenic pathways AKT, TGF and MAPK (Figure 1.), which is in agreement with observations that the density of endothelium decreases as the drusen size increases [43]. It probably happens due to decreased permeability of oxygen and nutrients through the Brush membrane, which results in hypoxia in the sub-RPE region. There is a close relationship between the RPE and choroid, where the loss of RPE affects the integrity of choroid and vice versa [44]. Recent studies [45] suggest loss or dedifferentiation of choroidal endothelial cells before loss of the RPE in eyes with AMD. One possible stimulus for angiogenesis is hypoxia. Hypoxia-inducible factor (HIF) is a transcription factor that undergoes dehydroxylation and deacetylation, modifications preventing it from ubiquitin degradation, due to hypoxia. Due to hypoxia, HIF translocates to the nucleus and induces expression of secreted growth factors (VEGF, FGF, and TGF) which promote signaling pathways p38, AKT, ERK, JNK, TGF and MAPK (Figure 1.) which leads to cell migration and proliferation, and to increased vascular permeability during AMD. While endothelial genes show decreased expression, we observed activation of pro-angiogenic, proliferation pathways which clearly demonstrates compensatory needs for oxygen supply in AMD affected tissues. Up regulation of IGF2, whose product up-regulates VEGF expression [46], can serve as an additional source of TGFbeta pathway activation [47].

### Elevated cellular immune response

Elevated cellular immune response is associated with almost all AMD phenotypes - similar results were obtained in this study for the chemokine pathway, which reflects the common nature of AMD disease. Activation of the cell-mediated immune pathway was confirmed by Gene Ontology analysis which revealed increased expression of all known CXCR3 ligands and pro-inflammatory chemokines: CXCL1, CXCL2, CXCL9, CXCL10, CXCL11, CCL2, and CCL8 in the RPE-choroid [48]. Sets of these chemokines are able to launch a full immune response via activation of macrophages, dendritic cells, granulocytes, CD4+ Th1 cells, CD8+ T cells, and natural killer cells in disease affected tissue [49]. Also we found increased activation of the inflammation pathway IL-2 which is fully consistent with conclusions drawn from clustering analysis of several AMD phenotype-specific RPE-choroid modules that inflammation is a prevalent functional category [41]. IL6, presumably a mediator of RPE degeneration, is also significantly increased upon AMD progression [50] and could account for activation of STAT3, ERK and AKT signaling pathways. Enhanced level of IL1B found in the RPE-choroid tissue of AMD samples can contribute to strong p38 pathway activation. Altogether, these findings reveal

### Decreased apoptotic signaling

Interestingly, apoptosis-related pathways showed decreased activity in AMD samples in our study, which may be due to alternative mechanisms of non-apoptotic cell death like necrosis or autophagy. In our research we did not stratify our samples depending on different AMD types/stages, as we wanted to perform a direct comparison of two independent experiments and AMD stage stratification was present only in one data set (GSE29801), but not in GSE50195. However, according to the authors of the GSE29801 data set, there is detectable upregulation of apoptosis-related transcripts in the geographic atrophy (GA) and CNV types of AMD [41]. This discrepancy could be explained by the assumption that in GA and CNV, apoptosis is a major way of cell death, while in the other AMD types, non-apoptotic mechanics of cell death are likely prevailing, and apoptosis pathways are suppressed. This assumption is supported by up regulation of the EPO gene and erythropoietin pathway during AMD, which are found to inhibit oxidative damage-induced apoptosis in cultured RPE cells [51].

### Newly-discovered pathway alterations

In addition to pro–angiogenic, pro-inflammatory and apoptotic pathways which were previously known to play important roles in AMD, we found several new pathways activated: Wnt, mTor, Glucocorticoid, cAMP, estrogen and androgen receptor signaling pathways. While biological roles of activation of these pathways needs to be further clarified, we can consider them to belong to a newAMD-specific pathway activation signature.

### Cluster and quality control analysis

**Figure 2:**
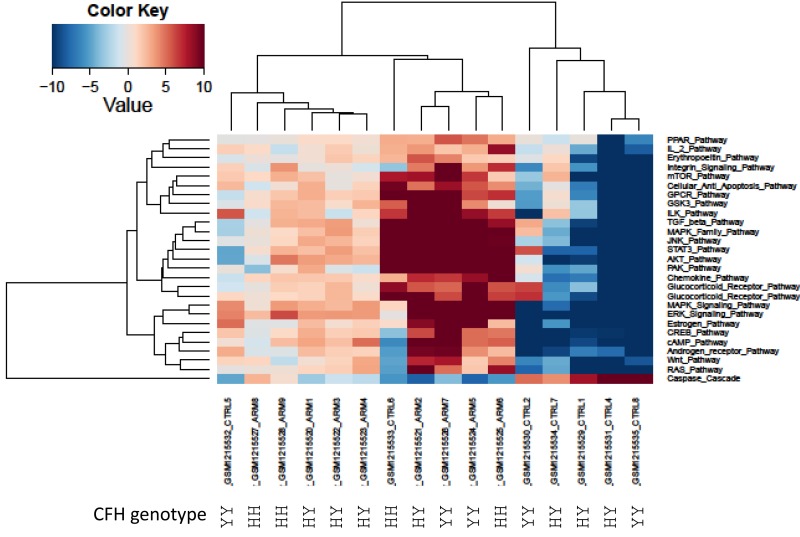
Heatmap of differentially activated pathways shown in Figure 1A Complement factor H genetic background (rs1061170 SNP) for PAS values derived from GSE50195 dataset shown for high-risk YH/HH and low-risk YY genotype. Blue shading indicates pathway downregulation; red shading indicates pathway upregulation. Samples with names ending in CTRL indicates control samples; samples with names ending in ARM indicates AMD samples.

Since AMD has several well-known genetic variations directly associated with high risk of AMD development, we investigated the genetic background available in one of the datasets (GSE50195). We used complement factor H genotype (rs1061170 SNP) [52] information to conduct cluster analysis with pathway activation values obtained from the GSE50195 dataset (Figure 2.) Our analysis revealed three major groups of samples: two groups of AMD and one group of control. Samples with low risk rs1061170 SNP variation, which was present only in two AMD samples, cluster together. These findings clearly demonstrate heterogeneity of the AMD cohort (Figure 2.).

**Figure 3:**
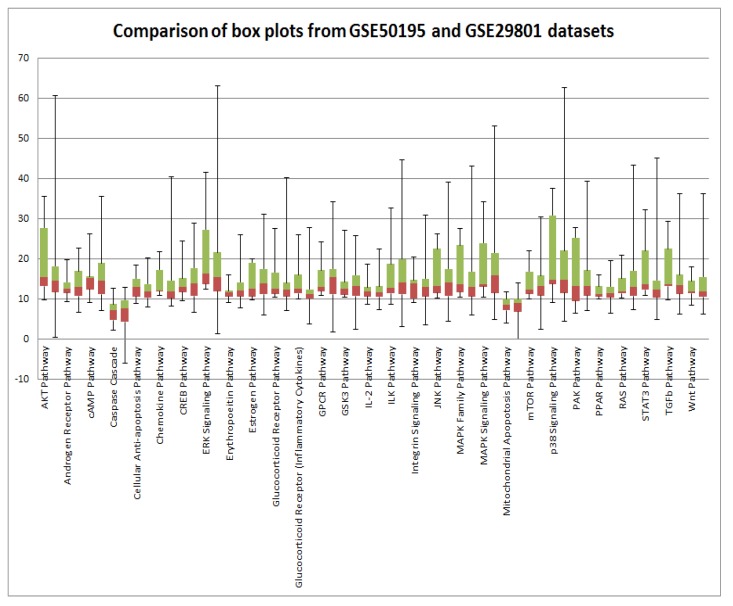
Comparison of GSE29801 derived PAS distribution and GSE50195 derived PAS distribution Box plots of GSE29801 (right) derived PAS and GSE50195 (left) derived PAS for each pathway. All PAS values for each pathway from two independent data sets are comparable; moreover box plots for GSE50195 derived PAS lay inside of box plots for GSE50195 derived PAS. Box plot whiskers represent min and max values for each pathways.

In order to validate our results, we analyzed two independent data sets obtained in two independent experiments. High variability of PAS values could be attributed to postmortem RNA degradation; despite all quality control measures it is very difficult to preserve all samples in the same way. An additional source of variability is different or misinterpreted AMD stage. There is still an open question regarding AMD disease etiology: is it a single multi symptom disease, or several diseases under the AMD manifestation umbrella? Such discrepancies could introduce a huge source of variability. Mainly to address these issues, we used data from two independent microarray experiments. Despite high variability of PAS values for almost all pathways for each data set, box plots perfectly illustrate the strong similarity of PAS values between both data sets (Figure 3). Transcriptional heterogeneity in human donor eye tissue can arise from multiple factors, including normal genetic variation, environmental influences unrelated to AMD, the presence of mixed cell types, and/or variable degrees of AMD progression.

## CONCLUSION

In this study, we discovered new pathways activated during AMD disease: Wnt, mTor, glucocorticoid, cAMP, estrogen and androgen receptor signaling pathways. Further studies will be required to figure out which cell populations are responsible for which individual pathway activation changes. Better understanding of the intimate interplay of signaling pathway activation in normal aging and AMD may uncover new diagnostic and treatment options [53-55] and studies of the various geroprotective drugs may lead to new preventative strategies [56].

### Towards system-level pathway integration

**Figure 4:**
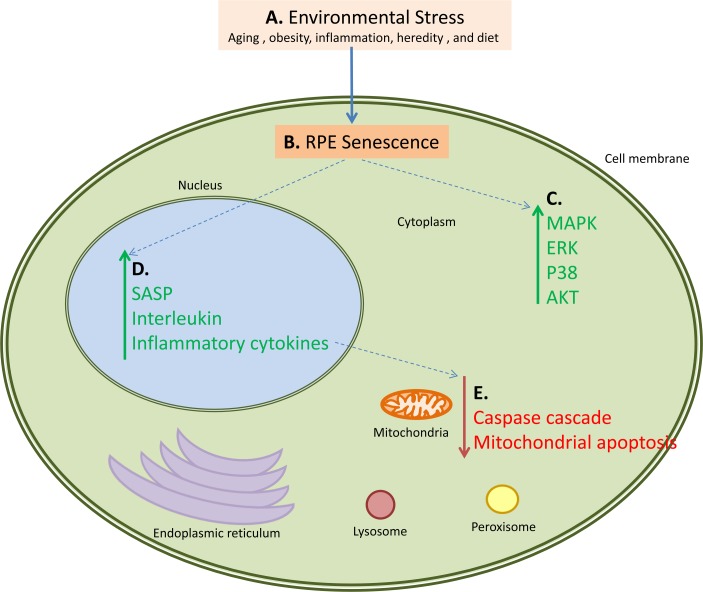
An example of how multiple pathways are activated and down-regulated during AMD This figure also serves as a working hypothesis for the pathogenesis of AMD. Proposed steps and interactions are as follows: A. Environmental Stress in the form of aging, obesity, inflammation, or diet causes B. senescence and loss of proliferation of the retinal pigment epithelial cells leading to C. activation of the MAPK, ERK, p38 and AKT pathways in the cytoplasmic components of the cells. This cellular senescence also has several consequences, primary of which are D. upregulation of the SASP, interleukin, and inflammatory cytokine networks, and E. downregulation of the caspase cascade and mitochondrial apoptosis. These pathways also interact; for example the upregulation of the SASP, interleukin, and inflammatory cytokine pathways causes downregulation of the caspase and mitochondrial apoptosis pathways. Green arrows represent upregulated pathways, red arrows represent downregulated pathways, and blue dotted arrows represent connected pathways.

Our results represent an advance toward better understanding of systems-level changes in pathway activation responsible for transition of normal tissues to the AMD phenotype (Figure 4). Also we demonstrated participation of immune, inflammatory and cell survival pathways in AMD progression that directly validates our method of interactome analysis.

Taking together already established facts and our new findings, we demonstrated that AMD is a single origin disease with multiple phenotypes, and which has an aging related lipid metabolism dysregulation as a starting point, followed by simultaneous aberrant activation of immune and inflammatory signaling pathways leading to a state of chronic local inflammation, followed by degeneration and neovascularization of affected tissues. The key closely-related questions are: what causes the first pathological changes in AMD, and does activation of signaling pathways observed in the current research represent a consequence or cause of AMD?

### Future research

Our approach of transforming gene expression data into signaling pathway activation strength profiles reveals many attractive targets for AMD therapeutics and diagnostics (Figure 4). Along with pharmaceuticals targeting, core immunological processes, inhibitors of pathways activated in AMD may have potential efficacy for prevention or treatment of some clinical manifestations of this disease.

## METHODS

### Source datasets

In this study, we utilized microarray gene expression data taken from two data sets found in NCBI GEO (http://www.ncbi.nlm.nih.gov/geo/query/acc.cgi?acc=GSE50195, http://www.ncbi.nlm.nih.gov/geo/query/acc.cgi?acc=GSE29801) database in order to examine pathways that are affected by AMD. First dataset GSE50195 contains the information on 16 samples from RPE-Choroid tissues profiled using Affymetrix Human Exon ST 1.0 arrays: 9 samples from AMD affected tissues and 7 from normal tissues [39]. Second dataset GSE29801 contains the data for 172 RPE-choroid and 121 retina samples from normal and AMD human donor eyes (31 normal eyes and 37 eyes at different stages of AMD disease.) profiled using Agilent-014850 Whole Human Genome Microarray 4×44K. We used only RPE-choroid samples from GSE29801 data set: 94 samples from normal tissues and 78 samples from AMD affected tissues [41].

### Functional annotation of gene expression data

For the functional annotation of the primary microarray gene expression data, we applied our original algorithm termed OncoFinder [27]. It enables calculation of the Pathway Activation Strength (PAS), a value which serves as a qualitative measure of pathway activation. This algorithm has been also shown to minimize error in the comparisons of data obtained using different experimental platforms [27]. The signaling pathways knowledge base developed by SABiosciences (http://www.sabiosciences.com/pathwaycentral.php) was used to determine structures of intracellular pathways, which were used for OncoFinder as described previously [27, 28]. Using R Bioconductor package, raw microarray values were subject to background subtraction and quantile normalization according to [57]. Values of gene expression for all samples were combined in single file which had been used as an input for AMD Medicine pathway analysis software. This software suite is a cloud based implementation of Oncofinder algorithm [27], optimized for AMD studies. Briefly, the algorithm utilizes the following formula to evaluate pathway activation:

$$PAS_{p}=\sum_{n}ARR_{np}\;.\;BTIF_{n}\;.\;{\text{lg(CNR}}_{n})$$

Here the *case-to-normal ratio*, *CNRn*, is the ratio of expression levels for a gene *n* in the sample under investigation to the same average value for the control group of samples. The Boolean flag of *BTIF* (*beyond tolerance interval flag*) equals to zero when the *CNR* value has passed simultaneously the two criteria that demark the significantly perturbed expression level from essentially normal: first, the hybridization signals for the sample lie within the tolerance interval, where p-value>0.05, and second, the value of *CNR* differs from 1considerably, *CNR* 0.66 or *CNR* 1.5. The discrete value of *ARR* (*activator / repressor role*) equals to thefollowing numbers: −1, when the gene/protein *n* is a repressor of pathway excitation; 1, if the gene/protein *n* is anactivator of pathway excitation; 0, when the gene/protein *n* can be both an activator and a repressor of thepathway; 0.5 and −0.5, respectively, if the gene/protein *n* is rather an activator or repressor of the signalingpathway *p*, respectively. Results for the 273 pathways were obtained for each sample (listed in Supplemental Table_GSE50195_AMD.xlsx and Table_GSE29801_RPE_AMD.xlsx). Statistical tests were done with the MS Excel software. Heatmap generation and hierarchical clustering were performed using R v3.1.0.

## SUPPLEMENTAL TABLES


