# Supplementary figures and images for: Pathway activation profiling reveals new insights into Age-related Macular Degeneration and provides avenues for therapeutic interventions
Source: Aging (Albany NY). 2014 Dec 22;6(12):1064–75. doi: 10.18632/aging.100711 (PMC4298366; doi:10.18632/aging.100711)

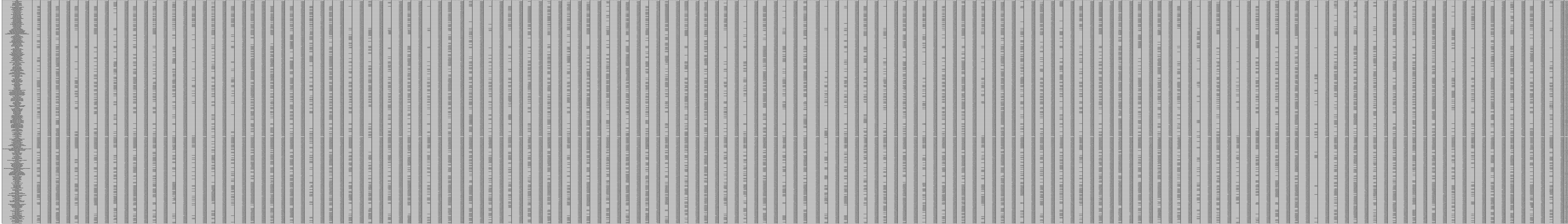

Supplement: Supplementary file 2 [file aging-06-1064-s002.pdf]
