# Supplementary material for: Pathway activation profiling reveals new insights into Age-related Macular Degeneration and provides avenues for therapeutic interventions
Source: Aging (Albany NY). 2014 Dec 22;6(12):1064–75. doi: 10.18632/aging.100711 (PMC4298366; doi:10.18632/aging.100711)
Supplement: Supplementary file 1 [file aging-06-1064-s001.pdf]

	GSM1215520_ARM1	GSM1215520_ARM1_p-value	GSM1215521_ARM2	GSM1215521_ARM2_p-value	GSM1215522_ARM3	GSM1215522_ARM3_p-value	GSM1215523_ARM4	GSM1215523_ARM4_p-value	GSM1215524_ARM5	GSM1215524_ARM5_p-value	GSM1215525_ARM6	GSM1215525_ARM6_p-value	GSM1215526_ARM7	GSM1215526_ARM7_p-value	GSM1215527_ARM8	GSM1215527_ARM8_p-value	GSM1215528_ARM9	GSM1215528_ARM9_p-value	p-value_Mean
Pathway																			
0_IL_6_Pathway_IKBs_Degradation	0	0.407736365	0	0.407736365	0	0.407736365	0	0.407736365	0.58835448	0.259874406	0	0.407736365	1.555669519	0.104591167	0	0.407736365	0	0.407736365	0.525358404
AHR_Pathway	0	0.348461469	0	0.348461469	0	0.348461469	0	0.348461469	0	0.348461469	0	0.348461469	-0.408462005	0.442878104	0	0.348461469	-0.987999161	0.41757159	0.791296
AHR_Pathway_AHR_Degradation	0	0.378688377	0	0.378688377	0	0.378688377	0	0.378688377	0	0.378688377	0.969285713	0.152593804	0	0.378688377	0	0.378688377	0	0.378688377	0.266379923
AHR_Pathway_C_Myc_Expression	0	0.356963834	0	0.356963834	0	0.356963834	0	0.356963834	0	0.356963834	0	0.356963834	-0.408462005	0.487728146	0	0.356963834	-0.987999161	0.328203364	0.873844699
AHR_Pathway_Cath_D_Expression	0	0.356963834	0	0.356963834	0	0.356963834	0	0.356963834	0	0.356963834	0	0.356963834	-0.408462005	0.487728146	0	0.356963834	-0.987999161	0.328203364	0.873844699
AHR_Pathway_PS2_Gene_Expression	0	0.356963834	0	0.356963834	0	0.356963834	0	0.356963834	0	0.356963834	0	0.356963834	-0.408462005	0.487728146	0	0.356963834	-0.987999161	0.328203364	0.873844699
AKT_Pathway	3.539448713	0.238338347	14.44078166	0.119459851	3.063238394	0.245057788	2.64071067	0.251126836	25.35859459	0.056354596	17.63413536	0.096219595	20.36729634	0.079730789	-0.684433509	0.302280783	4.774933311	0.221507821	0.006950509
AKT_Pathway_Aggregation_&_Neurodegeneration	0	0.477028799	-1.068310897	0.152671965	0	0.477028799	0	0.477028799	0.115154707	0.479251235	0	0.477028799	-0.597913321	0.268811168	0	0.477028799	-0.632281646	0.258672963	0.711025407
AKT_Pathway_Apoptosis_Inhibition	0	0.460370182	-1.068310897	0.206924788	0	0.460370182	0	0.460370182	0.115154707	0.492218356	0	0.460370182	-0.597913321	0.30531321	0	0.460370182	-0.18278955	0.410571135	0.873844699
AKT_Pathway_Blocks_Apoptosis	0	0.416920138	-1.068310897	0.142566926	0	0.416920138	0	0.416920138	-0.483170545	0.269682745	0	0.416920138	-0.787779226	0.195868504	0	0.416920138	-0.632281646	0.231469616	0.185788722
AKT_Pathway_Cardiovascular_Homeostasis	0	0.460370182	-1.068310897	0.206924788	0	0.460370182	0	0.460370182	0.115154707	0.492218356	0	0.460370182	-0.597913321	0.30531321	0	0.460370182	-0.632281646	0.297287719	0.791296
AKT_Pathway_Caspase_Cascade	0	0.470005029	-1.068310897	0.153861717	0	0.470005029	0	0.470005029	0.115154707	0.487293444	0	0.470005029	-1.482205563	0.090172786	0	0.470005029	-0.632281646	0.257516748	0.397101146
AKT_Pathway_Cell_Cycle	0	0.42576924	0.518811375	0.196483448	0	0.42576924	0	0.42576924	0	0.42576924	0	0.42576924	1.412702553	0.036581759	0	0.42576924	0.632281646	0.16108037	0.314614455
AKT_Pathway_Cell_Cycle_Progression	0	0.425979482	-1.068310897	0.138949028	0	0.425979482	0	0.425979482	0.115154707	0.466663413	0	0.425979482	-1.037085668	0.144337556	0	0.425979482	-0.632281646	0.230993659	0.491432025
AKT_Pathway_Cell_Survival	0	0.36788243	0	0.36788243	0	0.36788243	0	0.36788243	0	0.36788243	0	0.36788243	-1.195240223	0.000806244	0	0.36788243	-0.632281646	0.017607063	0.711025407
AKT_Pathway_Death_Genes	0	0.446455575	-1.068310897	0.145912593	0	0.446455575	0	0.446455575	0.115154707	0.488128514	0	0.446455575	-1.037085668	0.151625715	0	0.446455575	-0.632281646	0.243281147	0.397101146
AKT_Pathway_Elevation_of_Glucose_Import	0	0.446455575	-1.068310897	0.145912593	0	0.446455575	0	0.446455575	0.115154707	0.488128514	0	0.446455575	-0.547073056	0.266808048	0	0.446455575	-0.632281646	0.243281147	0.525358404
AKT_Pathway_ERK_Pathway	0	0.416920138	-1.068310897	0.142566926	0	0.416920138	0	0.416920138	-0.483170545	0.269682745	0	0.416920138	-0.787779226	0.195868504	0	0.416920138	-0.632281646	0.231469616	0.185788722
[illegible]	[illegible]	[illegible]	[illegible]	[illegible]	[illegible]	[illegible]	[illegible]	[illegible]	[illegible]	[illegible]	[illegible]	[illegible]	[illegible]	[illegible]	[illegible]	[illegible]	[illegible]	[illegible]	[illegible]
